# Environmental Niche Dynamics of Blue Grama (*Bouteloua gracilis*) Ecotypes in Northern Mexico: Genetic Structure and Implications for Restoration Management

**DOI:** 10.3390/plants11050684

**Published:** 2022-03-02

**Authors:** Carlos R. Morales-Nieto, Federico Villarreal-Guerrero, Pedro Jurado-Guerra, Jesús M. Ochoa-Rivero, Jesús A. Prieto-Amparán, Raúl Corrales-Lerma, Alfredo Pinedo-Alvarez, Alan Álvarez-Holguín

**Affiliations:** 1 Facultad de Zootecnia y Ecología, Universidad Autónoma de Chihuahua, Periférico Francisco R. Almada km. 1, Chihuahua 31453, Mexico; cnieto@uach.mx (C.R.M.-N.); fvillarreal@uach.mx (F.V.-G.); jamparan@uach.mx (J.A.P.-A.); rclerma@uach.mx (R.C.-L.); apinedo@uach.mx (A.P.-A.); 2 Instituto Nacional de Investigaciones Forestales, Agrícolas y Pecuarias (INIFAP), Campo Experimental La Campana, Carretera Chihuahua-Ojinaga km. 33.3, Aldama 32190, Mexico; jurado.pedro@inifap.gob.mx (P.J.-G.); ochoa.jesus@inifap.gob.mx (J.M.O.-R.)

**Keywords:** environmental niche modeling, genetic diversity, MaxEnt, molecular markers, restoration

## Abstract

Understanding the genetic structure adopted by natural populations and its relation to environmental adaptation is critical for the success of restoration programs. We evaluated the genetic structure and temporal environmental niche dynamics of blue grama (*Bouteloua gracilis*) in 48 populations. The genetic evaluation was performed through amplified fragment length polymorphism (AFLP) molecular markers. The maximum entropy method was used to model the past, present, and future environmental niches of the three clusters derived from the genetic analysis. The environmental niches of the three genetic clusters showed dynamic overlaps and isolations during the last interglacial and glacial maximum. The paleoclimatic events, which occurred during those periods, may have reinforced genetic exchange among populations and affected their genetic structure. Genetic clusters also presented different environmental niches in the present. Thus, they can be considered as three distinct ecotypes and restoration programs must be carried out using local germplasm from each environmental niche to increase their chance of success. Based on the environmental niches of the genetic clusters, changes are expected in the near and mid-century future. Therefore, climate change must be considered for species conservation management and future restoration programs.

## 1. Introduction

The assessment of genetic structures provides insights into genetic differences within species occurring in response to environmental adaptation pressures. Knowledge about the genetic structure allows researchers to quantify the number of genotypes within species and determine their geographical distribution. This information may be useful for modeling the migration patterns shown by different genotypes through time. It may also be useful to determine the interactions that may have influenced the adaptative genetic differentiation within species [1].

Environmental niche models are statistical and mathematical models used to infer and predict the geographic distribution of species [2]. Although modeling at the genotype level is not infrequent, most environmental niche models (ENMs) have been conducted at the species level, assuming all of the populations exhibit the same environmental adaptation. However, genotypes within species often exhibit local adaptation and distinct responses to environmental conditions [3,4]. Therefore, the incorporation of genetic structure information into ENMs may improve the accuracy of the niche predictions [5,6,7].

Several recent studies have incorporated genetic structure information into environmental niche modeling to obtain accurate predictions and support genetic conservation strategies [8,9,10,11,12,13]. This approach is known as genetically informed environmental niche models (gENMs) [6]. Such models may help to increase the probability of the success of restoration programs, since they allow the identification of suitable areas for the species genotypes [14,15]. Thus, knowledge about the genetic structure and gENMs are important to design strategies for the conservation and utilization of native germplasm.

One of the species most utilized for grasslands restoration in North America is blue grama (*Bouteloua gracilis* (Kunth) Lag. ex Griffiths). This grass has been widely used due to its proven adaptability to a wide range of environmental conditions and its high forage quality [16,17]. In the past years, several breeding programs have been performed to select outstanding genotypes of this species that may be employed to restore degraded grasslands [17,18,19,20]. These selection programs have mainly focused on agronomic traits, such as high biomass production, while the genetic structures and environmental niches of the selected genotypes have not been considered as selection factors.

The maximum entropy method has been widely used to estimate the environmental niches of grass species [21,22]. This method is available in the MaxEnt model, which is a presence-only algorithm used to infer species distribution through an environmental niche modeling approach [23]. MaxEnt has several advantages, such as the possibility of obtaining a high level of certainty using limited records of presence [24]. This model has been used at the species level to identify suitable areas for the distribution and use of blue grama in Mexico and the United States [25,26]. Hence, it may serve for modeling the environmental niche of the blue grama genotypes. 

This study addressed the following questions: first, do the blue grama populations from northern Mexico have a special genetic structure pattern that can be split into genetically distinct clusters? Second, did the paleoclimatic events of the last interglacial and glacial maximum affect the gene exchange and the genetic structure of the blue grama populations? Third, do the genetic clusters have different present environmental niches? Fourth, will future climate change differentially affect the environmental niches of blue grama genetic clusters?

## 2. Results

### 2.1. Genetic Structure

**Figure 1:**
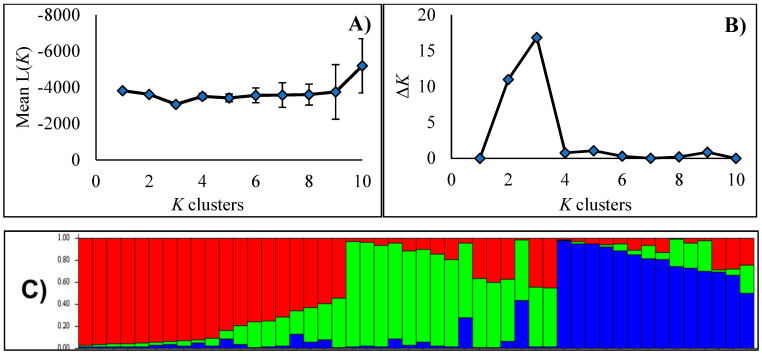
Genetic structure of 48 blue grama (*Bouteloua gracilis*) populations from Chihuahua, Mexico, inferred from AFLP data and STRUCTURE analysis. (**A**) Mean log-likelihood (*L*(*K*)) and (**B**) Evanno’s delta *K* (ΔK) values. *K* values tested were from 1 to 10. (**C**) Bar plots representing the estimated membership probability (y-axis) of a population to belong to a specific cluster (indicated by specific color).

The AFLP primer combinations generated 186 fragments and 67.7% of these fragments were polymorphic. Results from the STRUCTURE analysis indicated that *K* = 3 was the number of genetic clusters with the strongest support, since it obtained the highest L(*K*) and Δ*K* values (Figure 1).

**Figure 2:**
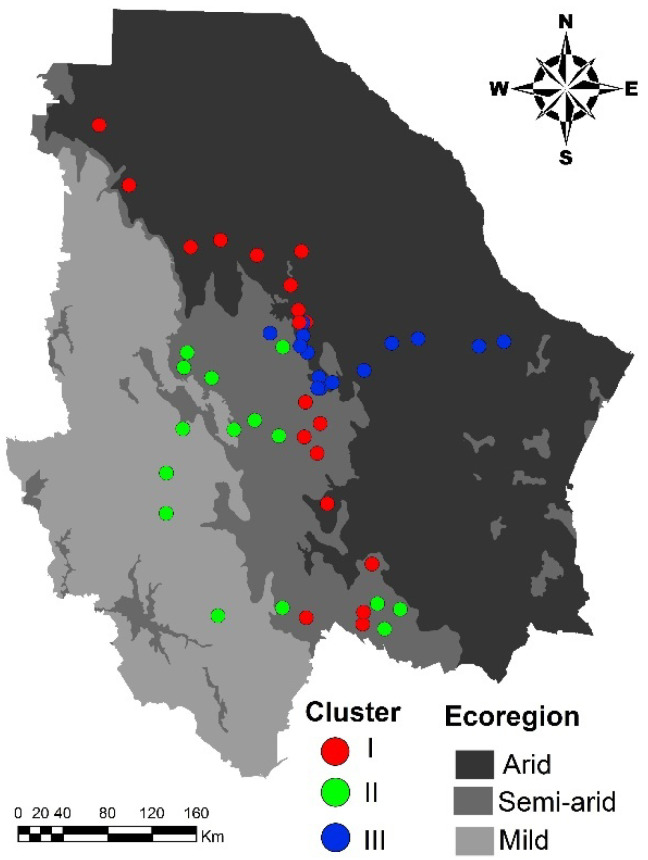
Genetic structure of 48 blue grama (*Bouteloua gracilis*) populations in a geographical context, split into three clusters by AFLP based STRUCTURE analysis.

The genetic structure of the blue grama populations may have been generated by an evolutionary adaptation to the environmental conditions prevailing in Chihuahua, since an association among the ecoregions of the state and the genetic clustering pattern was found (chi-square = 29.2; *p* < 0.0001). The populations belonging to Cluster I are located in the semi-arid (42.1%) and arid (57.9%) regions of the state. Populations from Cluster II are located in the semi-arid (73.3%) and mild (26.7%) regions, while most of the populations (92.9%) integrating Cluster III are from the arid region (Figure 2).

The AMOVA revealed significant differences among the genetic clusters (*p* < 0.0001). Such differences only explained 16.6% of the total genetic variation and the majority of it (83.3%) exists within clusters. Accordingly, results from the AFLP analysis suggested a gene flow (Nm) value of 1.25 and an *Fst* of 0.167. These values indicate there is a relatively high genetic exchange and a low differentiation among genetic clusters. In addition, the diversity statistics showed that Cluster III exhibited the highest (*p* < 0.05) genetic diversity compared to the other genetic clusters (Table 1).

### 2.2. Environmental Niche of the Genetic Clusters

The MaxEnt model was used to predict the potential distribution of the three genetic clusters. A total of 19 sampling locations for Cluster I, 15 for Cluster II, and 14 for Cluster III were used for the environmental niche modeling, corresponding to the number of populations grouped in each genetic cluster. The AUC values were 0.83 ± 0.09, 0.85 ± 0.09, and 0.91 ± 0.06 for clusters I, II, and III, respectively. In addition, the three models obtained values higher than 0.5 in the null models. The mean diurnal range, mean temperature of the driest quarter, mean temperature of the warmest quarter, precipitation seasonality, and precipitation of the driest quarter were variables of great contribution in the ENMs of the genetic clusters (Table 2).

**Figure 3:**
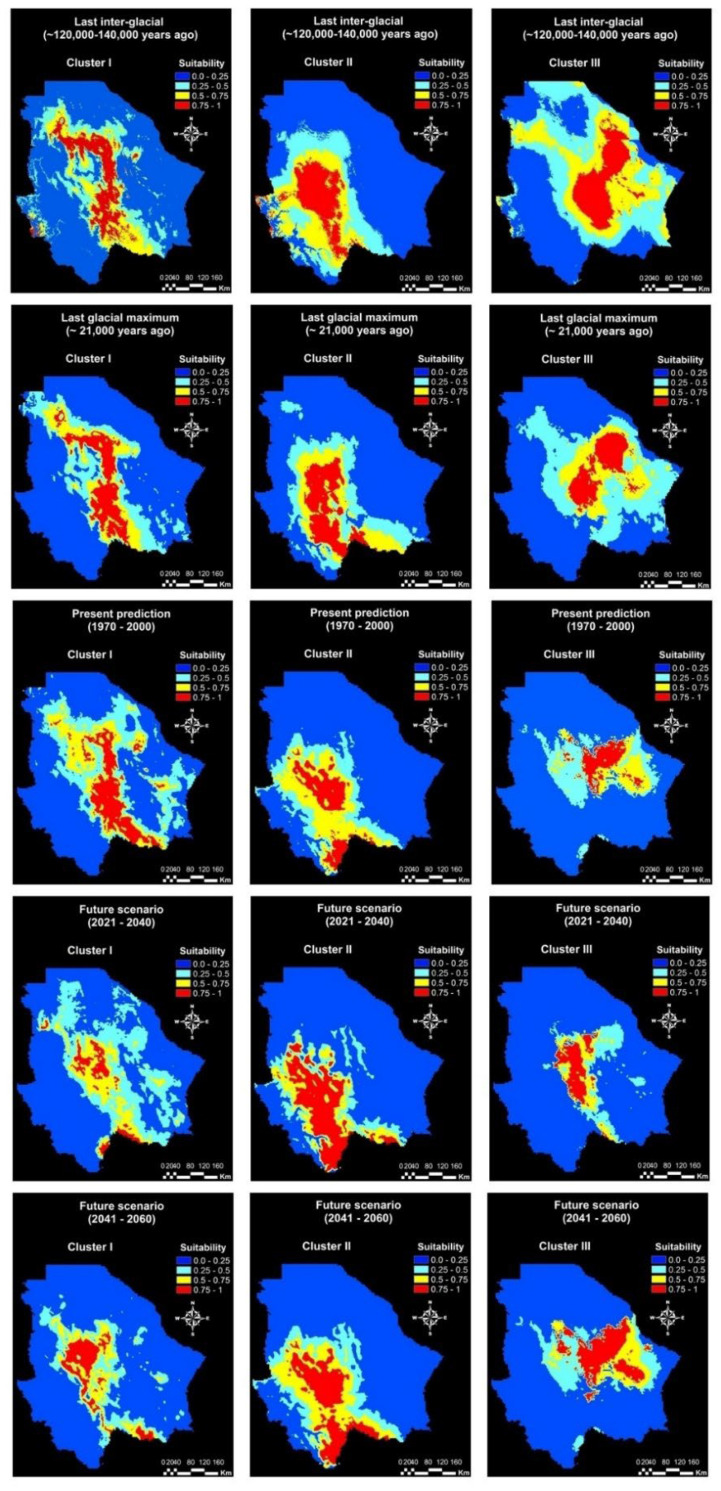
Potential distributions of three genetic clusters of blue grama (*Bouteloua gracilis)* in five different periods in Chihuahua, Mexico. Maps represent the MaxEnt niche model projections for the last interglacial, last glacial maximum, present, near future, and mid-century scenarios. The scale corresponds to the probability of habitat suitability.

The ENMs revealed quite different potential distribution ranges among the three genetic clusters in the past, present, and future scenarios (Figure 3). The areas with the highest probability of distribution (>0.75) of the three genetic clusters were quantified for a more accurate comparison of their probable distribution range. The surface with a high probability of habitat suitability (>0.75) of the three clusters varied over the different periods. Compared to the present ENM, the surface with a high probability of habitat suitability for Cluster I was smaller during the LIG and is expected to be smaller in the mid-century future. In contrast, Clusters I and II had a bigger surface with a high probability of habitat suitability during the LIG and LGM, and this surface is expected to be broader compared to the present ENM (Table 3). The statistic for niche overlaps revealed significant (*p* < 0.05) differences among the present ENMs of the three clusters (Table 4*).* The degree of niche overlapping among genetic clusters was similar in the past and present models. Nevertheless, the niche overlapping of Cluster III with the rest of the clusters is expected to be lower in the future.

**Figure 4:**
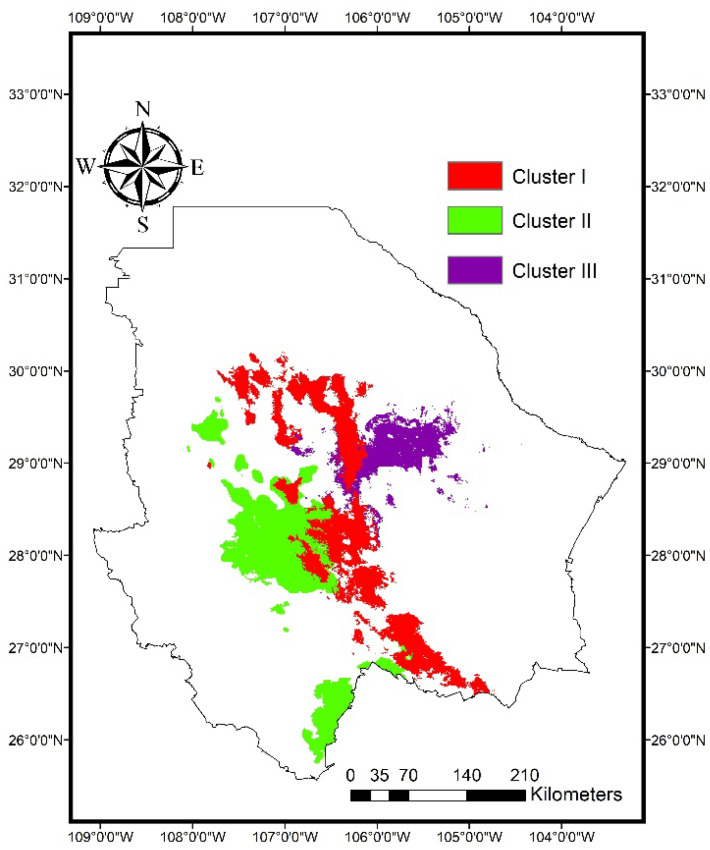
Areas with a probability of habitat suitability greater than 75% for the distribution of three genetic clusters of blue grama (*Bouteloua gracilis*) based on historical (1970–2000) bioclimatic variables, as derived from environmental niche models in MaxEnt.

Figure 4 shows the areas with a probability of habitat suitability greater than 75% for the distribution of the three genetic clusters at the present time. The areas with a high probability of habitat suitability (>0.75) varied among genetic clusters, and each genetic cluster occupied a different geographic range. The surface with a probability >75% for the distribution of Cluster I is mainly located in the central and southern part of the state, corresponding to the semi-arid region. Regarding Cluster II, the surface with a probability >75% is mainly distributed in the southwestern part of the state, close to the Sierra Madre Occidental. Finally, the surface with a probability >75% for Cluster III is distributed from the central to the eastern part of the state, mainly in the arid region (Figure 4).

**Figure 5:**
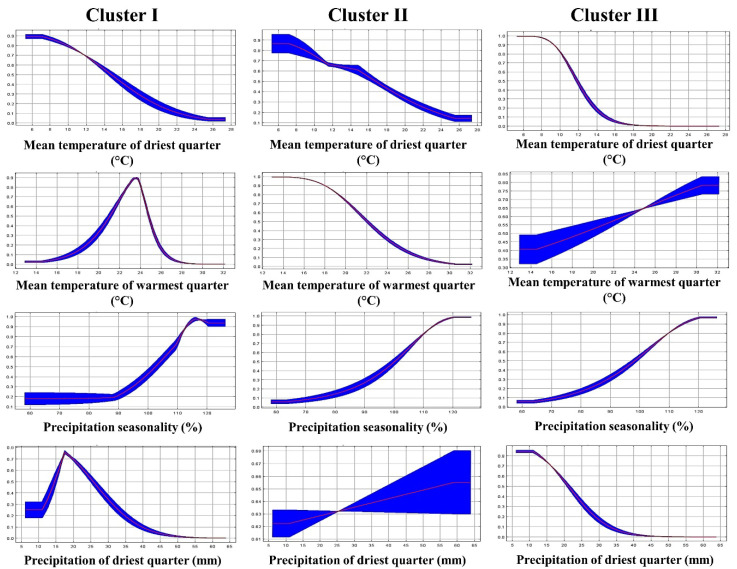
Response curves of the variables with the greatest contributions in the environmental niche models of three genetic clusters of blue grama (*Bouteloua gracilis*).

Figure 5 shows the response curves of some of the variables with the greatest contributions in the present ENMs of the genetic clusters. The curves revealed that the maximum probability of habitat suitability for Cluster I is in locations with mean temperatures in the warmest quarter close to 23 °C. The maximum probability of habitat suitability for Cluster II occurs close to 13 °C, while Cluster III reaches the highest probability of habitat suitability close to 31 °C. Regarding the precipitation in the driest quarter, the maximum probabilities of occurrence were found at 18, 58, and 8 mm, for Clusters I, II, and III, respectively.

## 3. Discussion

### 3.1. Genetic Structure

According to the results of the genetic analysis, the blue grama populations of northern Mexico exhibit a genetic structure that can be split into three genetic clusters. Genetic structures in blue grama populations were previously reported by Tso et al. [29]; these researchers evaluated 44 populations and 5 cultivars of blue grama from the southwestern United States through cpDNA analysis and AFLP markers. They concluded that the blue grama populations from the Colorado Plateau exhibit a genetic structure that may partially be explained by the local environmental variability. This agrees with the findings of the present study, since an association between the environmental conditions of the state and the genetic clustering pattern was found.

Evolutionary processes, such as environmental adaptation, gene flow, habitat fragmentation, and population isolation determine a population’s genetic structure. Accordingly, the reason for the divergence among genetic clusters might be adaptation to the different ecoregions of the state, since an association between the ecoregions of the state and the genetic clustering pattern was found. The populations from Cluster I are located in the central part of the state, in the transition zone between the semi-arid and the arid regions, while populations from Cluster II are located in the mild and semi-arid regions. Finally, populations belonging to Cluster III are mainly located in the arid region. Therefore, populations from Cluster III are better adapted to areas with lower precipitation and higher temperatures than the populations from Clusters I and II. Meanwhile, populations from Cluster II may be better adapted to lower temperatures than the other clusters. These results agree with previous studies, which found that adaptation to arid environments can produce genetic divergences among lineages in plants [30,31,32].

The AMOVA partitioning of the genetic variation revealed there is a high level of genetic diversity within clusters, since a quite high partition (83.3%) of the variation exists within clusters. This suggests the possibility of selecting outstanding populations from each cluster, instead of only selecting them from all the populations evaluated.

Furthermore, the high level of diversity within clusters observed in this study agrees with patterns of genetic diversity found in other grass species. For example, Xiong et al. [33] analyzed the genetic diversity of the grass *Psathyrostachys junceain* and found that most of the variation (87.6%) occurred within populations. Similar results were found by Wu et al. [34], who reported that the genetic differences among populations of *Elymus tangutorum* explained 26.1% of the global variation. Likewise, Tso et al. [29] found that approximately 13% of variation was attributed to genetic differences among blue grama populations.

The low level of genetic differentiation among clusters suggests a high genetic exchange exists among the blue grama populations. Accordingly, the Nm among genetic clusters was 1.25, which can be considered high compared with those obtained for other grasses. For instance, Zhang et al. [31] found an Nm value of 0.95 among ten populations of the grass *Festuca ovina*. Similarly, Coppi et al. [35] reported an Nm of 0.49 among *Phragmites australis* populations. Furthermore, the *Fst* obtained in this study (0.167) revealed a relatively low differentiation among genetic clusters. Mitchell et al. [36] obtained an *Fst* of 0.02 among 85 Australian populations of *Microlaena stipoides*. In the study by Tso et al. [29], *Fst* values of 0.15 to 0.22 for 44 populations and 5 cultivars of blue grama were reported.

Cluster III obtained the highest values (*p* < 0.05) for all the diversity statistics evaluated. Populations integrated into this cluster are mainly distributed throughout the arid region, suggesting blue grama populations from dry habitats tend to have a high genetic diversity. This agrees with previous studies, which concluded that populations from adverse environments tend to have a high genetic diversity [37,38]. Accordingly, Zhao et al. [39] found that *Stipa krylovii* populations from drier environments tend to have a high genetic diversity as an evolutionary mechanism of adaptation to drought stress. Furthermore, previous research has reported a relationship between genetic diversity and environmental variables in grass species. For example, Zhang et al. [31] reported that Nei’s genetic diversity index correlates with the mean annual temperature (r = 0.56) and mean annual precipitation (r = −0.60). Similar results were also described by Zhang et al. [40] for *Dactylis glomerata*.

Cluster I, II, and III obtained *I* values of 1.38, 1.37, and 1.45, respectively. These values indicate a moderate level of genetic diversity within the clusters compared to those obtained in other studies. Wanjala et al. [41] obtained *I* values from 0.12 to 0.34 from an analysis of 281 cultivars of *Pennisetum purpureum*. Likewise, Todd et al. [42] analyzed 56 accessions of *Panicum virgatum* and reported an *I* of 0.317.

### 3.2. Environmental Niche Modeling

The incorporation of genetic structure information into the environmental niche modeling allowed us to obtain preliminary evidence of niche specialization among blue grama populations in northern Mexico. Past ENMs allowed researchers to recreate geographic distributions and revealed ancestral niche overlaps, which may have reinforced gene exchange between genetic clusters. They also revealed important patterns of genetic diversity and divergence related to environmental adaptation. Therefore, past ENMs with genetic structure information provided insight into the evolutionary processes, which have shaped the patterns of genetic diversity in blue grama. Such evolutionary processes included gene flow, environmental adaptation, habitat fragmentation, migration, and population isolation. These important patterns in the adaptative history of the species would be ignored by models at the species level.

Past ENMs revealed dynamic differences and overlaps among the niches of the genetic clusters during the LIG and LGM periods. During the LIG, the suitable areas for the distribution of Cluster II and III were 6000 and 6681 km^2^ respectively higher, than those in the present. However, the potential distribution of Cluster II increased in the mild region, while the potential distribution of Cluster III increased in the arid region. In North America, the climate was drier during the late LIG [43]. This was a consequence of the lower global ice volume, which produced higher sea levels, CO_2_ concentrations, and surface temperatures than the Holocene [44,45,46,47]. Therefore, Cluster III may be better adapted to drought and high temperatures than Clusters I and II, because its potential distribution increased to the arid region, showing better adaptability to the LIG warm and dry conditions. The niche overlaps among genetic clusters were higher during the LIG than in the present. Niche overlaps during the LIG may have reinforced gene exchange between genetic clusters and influenced the low genetic differentiation between them. This is consistent with earlier findings, which related genetic structure with paleoclimatic patterns [1,48].

During the LGM, Clusters I and III showed higher surface areas with a probability of habitat suitability >75% than during the LIG, while Cluster II showed a similar potential distribution. The niche overlapping between Clusters I and II was lower during the LGM than during the LIG. Meanwhile, the niche overlaps between Clusters I and III increased. These changes may have affected the gene flow among genetic clusters and their genetic structure. However, the three clusters showed higher surface areas with a probability of habitat suitability >75% during the LGM compared to the present potential distribution. The increase in the environmental niches of the blue grama genetic clusters is in agreement with the lower temperature and higher precipitation experienced during the LGM. The LGM was a glacial period characterized by a global cooling as a consequence of a reduction of the atmospheric CO_2_ and sea level [49,50]. The northern hemisphere faced a strong cooling, and northern Mexico was notoriously wetter than today due to increased rainfall events during the winter season [51,52].

In contrast, the present ENMs resulted in quite different geographical patterns among the three clusters. Clusters I and II have larger surface areas with a probability of habitat suitability >75% (7552 and 8854 km^2^, respectively) compared to Cluster III. This suggests that populations from Clusters I and II are adapted to a broader range of environmental conditions than Cluster III. Furthermore, the differences among the environmental niches of the three genetic clusters indicate they are three ecologically distinct units and can be considered as distinct ecotypes.

Given they are three distinct ecotypes, restoration programs with blue grama should be performed using local germplasms from the environmental niche of each cluster. This is consistent with previous studies, which indicate that the use of local germplasm may help to preserve the genetic diversity and increase the probability of the success of restoration programs [53,54]. However, most of the restoration projects with blue grama in Mexico have been performed using seed imported from the USA, which may be a factor limiting the establishment of this species. For this reason, several breeding programs have been performed to select outstanding genotypes of this species, which may be employed to restore degraded grasslands [17,18,19,20]. Nevertheless, these selection programs have been mainly focused on agronomic traits, such as high biomass production, while the genetic structures and environmental niches of the selected genotypes have not been considered as selection factors. Results from this study provide insight into the adaptability of blue grama populations in northern Mexico and highlight the potential of performing selection based on environmental adaptability. This information may be useful for future breeding programs.

Regarding the future scenarios, climate change will affect the suitable areas for the distribution of the genetic clusters. Future ENMs projected a broader surface area with a probability of habitat suitability >75% for Cluster I in the mid-century. Meanwhile, Cluster II will show a similar surface area and Cluster I will cover a smaller area. The increase in the potential distribution ranges of Cluster I would be associated with the severe droughts projected for North America in the mid-century as a consequence of anthropogenic drying [55,56]. Thus, populations from Cluster I appear to be more resistant to anthropogenic drying, since their potential distribution will increase towards the mid-century. In addition, climate change will modify the niche overlapping among the genetic clusters. The niche of Cluster III will experience the greatest changes by the mid-century. This may affect the genetic exchange among clusters and the genetic diversity of the blue grama populations from northern Mexico.

## 4. Materials and Methods

### 4.1. Population Sampling

**Figure 6:**
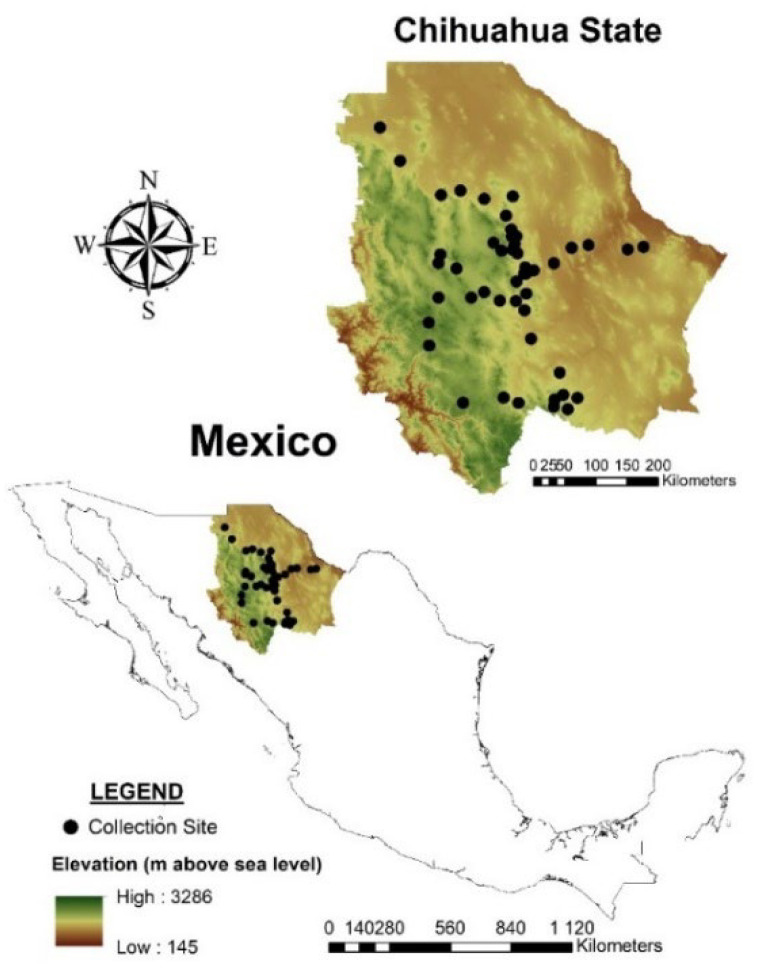
Geographic location of the collection sites of the 48 blue grama (*Bouteloua gracilis*) populations. Digital elevation model from INEGI (https://www.inegi.org.mx, accessed on 6 January 2022).

In this study, 48 natural populations were analyzed (Supplementary Materials Table S1; Figure 6). Populations were distributed across 29 municipalities of the state of Chihuahua, Mexico. Chihuahua borders the Mexican states of Durango, Coahuila de Zaragoza, Sonora, and Sinaloa. It also borders the North American states of Texas, New Mexico, and Arizona. This state is located in the geographic coordinates ranging from 31.78 to 25.55 N latitude and from −103.30 to −109.07 W longitude. It has a surface area of 247.4 km^2^, which represents 12.6% of the Mexican territory.

The state of Chihuahua was selected as the study area since it is representative of the species distribution in northern Mexico from both, the geographic and climatic perspectives. Sampling was performed in the temperate, arid, and semi-arid regions of the State. Populations were collected at least 20 km separated from each other, with the aim of gathering the greatest genetic diversity possible. The coordinates of each sampled site were recorded using a GPS Garmin eTrex 10, which exhibits a mean error value of 2.1 m. Fresh leaves from three plants were collected from each of the 48 populations and used for the genetic structure analysis. Collected leaves were stored in coolers and transported to the laboratory. Only three plants per population were analyzed, with the aim of assessing the greatest number of populations possible. To avoid sampling clones from rhizomes, the samples were taken from plants located at least 5.0 m apart. Sample sites were identified with a continuous number (N1, N2, N3, etc.) as they were collected. The plants from each population were also identified with a number from 1 to 3 (plant 1, plant 2, and plant 3).

### 4.2. Genetic Structure Analysis

The DNA extraction was carried out according to the method of Doyle and Doyle [57]. DNA from the three sampled plants from each population was bulked for the analysis. The genetic structure of the blue grama populations was evaluated based on the amplified fragment length polymorphisms (AFLP) approach, following Vos et al. [58]. The genomic DNA was digested using the restriction enzymes EcoRI and MseI, and ligated with EcoRI and MseI adapters. After the ligation restrictions, the resulting DNA was pre-amplified by adding an extra nucleotide (EcoRI + A and MseI + A). The pre-amplification products were used for the selective amplification, which involved the use of the following fluorescent-labeled primer combinations: MseI + CTG-EcoRI + AAG; MseI + CTG-EcoRI + ACT; MseI + CAG-EcoRI + AGG; MseI + CAG-EcoRI + AAC. The following amplification profile was applied for the polymerase chain reaction (PCR): 1 cycle of 30 s at 94 °C, 30 s at 65 °C, and 1 min at 72 °C; 12 cycles of 94 °C for 30 s, 65 °C for 30 s (with decrements of 0.78 °C at each cycle), and 72 °C for 1 min; and 23 cycles of 94 °C for 30 s, 56.8 °C for 1 min, and 72 °C for 1 min. The selective amplification products (2 μL) were mixed with 8 μL of formamide and 1 μL of LIZ 500 GeneScan (Applied Biosystems, Foster City, CA, USA) size standard. Primers fluorescently labeled at different wavelengths (700 nm and 800 nm) were added. The separation and selection of the amplified fragments were fulfilled on a DNA analyzer, LI-COR Model 4200. Finally, the presence or absence of the AFLP fragments was scored as “1” (presence of fragment) and “0” (absence of fragment) and transformed into a binary matrix for further analysis (Supplementary Materials Table S1).

The genetic structure was inferred using the model-based Bayesian clustering method, and implemented in the STRUCTURE software, ver. 2.3.4 [59,60]. This analysis was carried out using an admixed model with correlated allele frequencies for a range of possible genetic clusters (*K*) from 1 to 10. For each K, 30 independent replicates were conducted. A burn-in period of 10,000 and a run length of 100,000 Markov Chain Monte Carlo (MCMC) replications were implemented for each replicate. The optimal number of population genetic clusters was estimated based on the mean log-likelihood (L(K)) and the delta *K* (ΔK) statistics, using the Structure Harvester (http://taylor0.biology.ucla.edu/structureHarvester/, accessed on 6 January 2022) [61,62]. The relationship between the clustering patterns of the populations and the regions of the State was analyzed through a chi-square test of independence (α = 0.05).

An analysis of molecular variance (AMOVA) was performed to determine the genetic differentiation within and among the genetic clusters resulting from the STRUCTURE analysis [63]. This analysis was carried out based on PhiPT (analog of *FST*) using GenAIEx, ver. 6 [64]. Gene flow (Nm) among genetic clusters was calculated as Nm = (0.25 (1 − *FST*)/(*FST*)) [65].

For each genetic cluster, the diversity indices percentage of polymorphic loci, average of alleles per locus, average effective number of alleles per locus, Shannon information index (*I*), and Nei’s genetic diversity (*He*) were estimated using the software GenAIEx, ver. 6 [64]. The diversity indices among clusters were compared by using the Wilcoxon test with Bonferroni´s correction (α = 0.05).

### 4.3. Environmental Niche Modeling

The ecological niche modeling approach was applied to quantify the temporal ecological differentiation among the clusters derived from the genetic analysis. The past, present, and future suitable areas for the distribution of the genetic clusters were predicted based on the maximum entropy method using MaxEnt, ver. 3.4.4 [24,66]. The coordinates of the populations integrated into each genetic cluster were used as input for the ecological niche modeling. Initially, the 19 bioclimatic variables from the Worldclim database (https://www.worldclim.org) were obtained to perform the modeling [67]. Then, Pearson’s correlation test was applied to identify and exclude highly correlated variables (coefficient > 0.8). Only seven bioclimatic variables showed a degree of collinearity (Pearson’s correlation coefficient) lower than 0.8 in absolute values (positive or negative correlation). Only these seven variables were included in the models and they were: mean diurnal range (Bio2), temperature seasonality (Bio4), mean temperature of the driest quarter (Bio9), mean temperature of the warmest quarter (Bio10), mean temperature of the coldest quarter (Bio11), precipitation seasonality (Bio15), and precipitation of the driest quarter (Bio17).

The environmental niche models from the past were projected based on paleoclimatic data of the Last Interglacial (LIG; 120,000–140,000 years before present) and the Last Glacial Maximum (LGM; 22,000 years before present). The LIG bioclimatic layers were utilized at 2.5 arc-min resolution [50]. The LGM projections were performed using the Community Climate System Model (CCSM4) scaled down to a 30 arc-seconds resolution [68]. For the present potential distributions, bioclimatic grids derived from climatic data for the period 1970–2000 were utilized at 30 arc-seconds resolution.

The environmental niche models were also projected to future scenarios to assess how climate change will affect the potential distributions of the genetic clusters. The future bioclimatic variables with a 2.5 arc-minutes resolution were obtained from the MIROC-ES2L climatic model [69] for two periods: near future (2021–2040) and mid-century (2041–2060). The Representative Concentration Pathway (RCP) 4.5 was used as the expected climate change scenario, since it is a moderate emission scenario [70].

The models were constructed with a maximum number of interactions of 5000 and 10,000 background pseudo-absence points. The output format used was Cloglog. Most of the Maxent features were configurated as standard. Models were constructed by using 50 replicate runs, and the bootstrap method was set as the replicated run type. The models were built with 75% of sampling points, and the remaining 25% were randomly selected as test data. Fifty replicate runs were used to obtain a consensus model prediction and to calculate the area under the receiver operating curve (AUC) [71]. The model’s performance was evaluated based on the AUC and null models [72]. Models with AUC values greater than 0.75 were considered reliable while models with AUC values higher than 0.5 were considered null [73]. MaxEnt outputs were converted into geographic maps in ArcMap ver. 10.3 (ESRI, CA). The maps show the potential distribution range of the genetic cluster based on an index of suitability between 0 and 1, where 0 indicates that the environmental conditions are unsuitable and 1 indicates adequate conditions. The obtained environmental niches represent the modeled statistical association between the genetic structure and the environmental adaptation of the blue grama populations.

The niche identity test was performed to evaluate whether the genetic clusters would occupy different niches in the geographical space in the past, present, and future scenarios. The degree of niche overlapping existing between a pair of genetic clusters was quantified using the identity statistics Schoener’s D (*SD*) [27] and Warren’s I (*WI*) [28]. The *SD* and *WI* ranged from 0 to 1, where 0 indicates null overlap and 1 total overlap. To determine significance for the niche identity test, we ran 100 permutations in which sample labels were randomized to generate a null distribution of the identity statistics. If the empirical value of the statistics *SD* and *WI* fell below the 95% confidence interval of the null distribution, we considered the niches of the two clusters to be significantly different. The niche identity test was conducted through the ENM Tools available in the R software, ver. 4.0.4. 

## 5. Conclusions

The combination of genetic structure analysis and environmental niche modeling allowed us to quantify the major genetic lineages within blue grama populations from northern Mexico and predict their potential distribution over different periods. The blue grama populations analyzed in this study exhibit a special genetic structure pattern, and can be split into three distinct genetic clusters. The genetic structures of these lineages can be partially explained by the past paleoclimatic events of the LIG and LGM periods. The environmental niches of the three genetic clusters showed dynamic overlaps and isolations during the LIG and LGM, which may have affected the gene exchange among populations. Therefore, environmental adaptation may have contributed to shaping the genetic structure of the blue gamma populations during the history of the species.

Under the present scenario, each genetic cluster exhibits a different environmental niche. Hence, they can be considered as three genetically and ecologically distinct units, which means they are different ecotypes with different utilization potentials. Thus, restoration programs with blue grama in northern Mexico should be performed by using local germplasms from the environmental niche of each cluster.

According to the projections of future environmental niches, climate change will modify the suitable areas for the distribution of two of the genetic clusters. The potential distributions of Clusters I and III will be broader in the middle of the century, which suggests these populations are more resistant than populations from Cluster I to the anthropogenic drying projected as a consequence of climate change. Thus, climate change must be considered in species conservation management and future restoration programs. Finally, the high genetic diversity found within each cluster represents an opportunity to select outstanding germplasms, which may be used in future restoration programs.

## Figures and Tables

**Table 1:** Genetic diversity parameters within three population clusters of blue grama (*Bouteloua gracilis*).

Genetic Cluster	Percentage of Polymorphic Loci	Average Number of Alleles per Locus	Average Effective Number of Alleles per Locus	*I*	*H_e_*
Cluster I	63.4	1.59 ^b^	1.38 ^b^	0.333 ^b^	0.223 ^b^
Cluster II	66.6	1.61 ^b^	1.37 ^b^	0.337 ^b^	0.224 ^b^
Cluster III	73.1	1.67 ^a^	1.45 ^a^	0.389 ^a^	0.261 ^a^
Average	67.4	1.62	1.40	0.353	0.236

Different letters indicate significant differences among clusters (*p* < 0.05; Wilcoxon with Bonferroni´s correction); *I* = Shannon information index; *H_e_* = Nei’s genetic diversity.

**Table 2:** Percent contribution of seven bioclimatic variables to the environmental niche models for three genetic clusters of blue grama (*Bouteloua gracilis*).

ID	Variable	Cluster		
		I	II	III
Bio2	Mean diurnal range	1.4	18.1	2.3
Bio4	Temperature seasonality	2.0	11.5	0
Bio9	Mean temperature of the driest quarter	2.0	0.0	57.7
Bio10	Mean temperature of the warmest quarter	35.2	45.0	4.7
Bio11	Mean temperature of the coldest quarter	0.6	0.0	1.9
Bio15	Precipitation seasonality	44.0	25.4	8.4
Bio17	Precipitation of the driest quarter	14.7	0.0	25

**Table 3:** Suitable areas for three genetic clusters of blue grama (*Bouteloua gracilis*) in past, present, and future scenarios based on MaxEnt environmental niche models.

Environmental Niche Model	Genetic Cluster			Total
	Cluster I (km^−2^)	Cluster II (km^−2^)	Cluster III (km^−2^)	
Last inter-glacial (120,000–140,000 years ago)	13,257.3	22,962.2	14,789.5	53,009.00
Last glacial maximum (21,000 years ago)	18,506.70	22,887.1	17,668.4	54,062.2
Present prediction (1970–2000)	15,659.7	16,961.8	8107.6	40,729.2
Near future (2021–2040)	10,449.1	22,152.78	7968.9	40,570.7
Mid-century (2041–2060)	12,145.8	21,361.1	17,888.8	51,395.7

Surface areas with a probability of habitat suitability > 0.75.

**Table 4:** Niche identity statistics for three genetic clusters of blue grama (*Bouteloua gracilis*) in past, present, and future scenarios based on MaxEnt environmental niche models.

Environmental Niche Model	Cluster I vs. II		Cluster I vs. III		Cluster II vs. III	
	*SD*	*WI*	*SD*	*WI*	*SD*	*WI*
Last inter-glacial (120,000–140,000 years ago)	0.47 *	0.73 *	0.44 *	0.73 *	0.18 *	0.39 *
Last glacial maximum (21,000 years ago)	0.43 *	0.68 *	0.48 *	0.76 *	0.17 *	0.35 *
Present prediction (1970–2000)	0.43 *	0.68 *	0.42 *	0.69 *	0.18 *	0.33 *
Near future (2021–2040)	0.41 *	0.61 *	0.47 *	0.72 *	0.30 *	0.47 *
Mid-century (2041–2060)	0.43 *	0.72 *	0.24 *	0.51 *	0.13 *	0.25 *

*SD* = Schoener’s [27] and *WI* = Warren’s [28] statistic for niche overlap among clusters. Niche overlap, measured as Schoener’s D and Warren’s I, ranges from zero (no overlap) to one (niche models identical). * indicates significant differences (*p* < 0.05) among the environmental niches of the clusters.

## Data Availability

All the data presented in this study are available in the article and in Supplementary Materials.

## Supplementary Materials

The following are available online at https://www.mdpi.com/article/10.3390/plants11050684/s1, Table S1: Coordinates of the geographic location and AFLP binary data of the 48 blue grama (Bouteloua gracilis) populations.
